# Prevalence and correlates of adolescent pregnancy, motherhood and adverse pregnancy outcomes in Uttar Pradesh and Bihar

**DOI:** 10.1186/s12884-023-05354-6

**Published:** 2023-01-26

**Authors:** Neha Shri, Mayank Singh, Deepak Dhamnetiya, Krittika Bhattacharyya, Ravi Prakash Jha, Priyanka Patel

**Affiliations:** 1 Department of Survey research and Data analytics, International Institute for Population Sciences (IIPS), Mumbai, 400088 India; 2 Department of Fertility and Social Demography, International Institute for Population Sciences (IIPS), Mumbai, 400088 India; 3 Clinical Research Unit, AIIMS, New Delhi, Delhi, India; 4 Department of Statistics, University of Calcutta, Kolkata, 700019 India; 5 Department of Community Medicine, Dr Baba Saheb Ambedkar Medical College & Hospital, Delhi, 110085 India; 6 Department of Family and Generations, International Institute for Population Sciences (IIPS), Mumbai, 400088 India

**Keywords:** Adolescent, Pregnancy, Motherhood, Adverse pregnancy outcome, Uttar pradesh, Bihar

## Abstract

Pregnancy during adolescence is a major risk factor for adverse pregnancy outcomes. Further, Motherhood during the adolescent period is identified as a major global health burden. Considering the widely known importance of the negative impact of adolescent pregnancy, motherhood at an early age, and adverse pregnancy outcomes, this paper aims to provide insight into correlates of teen pregnancy, adolescent motherhood and adverse pregnancy outcome. This study utilizes the data from UDAYA survey conducted in Uttar Pradesh and Bihar. The eligible sample size for the study was 4897 married adolescent girls between the ages of 15 and 19 years. Bivariate analysis with a chi-square test of association and Multivariable logistic regression analysis was performed to fulfill the aim of the study. Our study shows that a major proportion of married adolescents (61%) got pregnant before the age of 20 years and around 42% of all adolescent married women gave birth to a child before reaching the age of 20 years. Adolescents who married before the age of 18 years were 1.79 times more likely to experience pregnancy (OR: 1.79; CI: 1.39–2.30) and 3.21 times more likely to experience motherhood (OR: 3.21; CI: 2.33–4.43). In the present study, women who experienced physical violence were at higher risk for having an adverse pregnancy outcome (OR: 1.41; CI: 1.08–1.84) than those who did not experience physical violence. To conclude, regional and national level efforts focused on improving early marriage, education and empowering women and girls can be beneficial.

## Introduction

Despite a substantial decline of 11.6% in adolescent-specific fertility rates in the past two decades globally, approximately 21 million girls in the age group 15–19 years become pregnant in developing countries [1]. Motherhood during adolescence period is regarded as a major global public health issue owing to the wide range of health effects and socio-economic consequences for the mother as well as the child. The World Health Organization estimates that about 11% of total births occurred to women aged less than 20 years, and 95% of these births occurred in low-and middle- income countries [2], usually among the most disadvantageous adolescents [3, 4]. Although the rights-based approach to health attempts to ensure that women have rights and control over their bodies and are free to decide on matters related to their sexuality and reproductive health, Child marriage, Early marriage and Forced Marriages (CEFM) is one of the most widespread practices that primarily affect girls and women. Early pregnancy is strongly influenced by the CEFM, which also raises the risks of complications during childbirth, such as miscarriage and maternal and newborn mortality [5].

Birth in teenage is a major risk factor for adverse pregnancy outcomes and has a significant negative impact on the future well-being of the infant as well as the mother [6, 7]. The adverse maternal and perinatal outcomes of adolescent pregnancy have been well-documented to be associated with low birth weight [8–10], preterm delivery [8, 10–12], perinatal death [13, 14], maternal death [11, 15] and neonatal asphyxia in low- and higher-income countries [12, 14]. India has the largest adolescent population in the world with 253 million adolescents aged 10–19 years. Estimates from the 4th National Family Health Survey indicate that 11.8 million teenage pregnancies occurred in India [16]. It is projected that by 2036, adolescents will constitute 23% & 16% of Bihar and UP population respectively [17]. The latest estimates indicate that 11% and three percent of women aged 15–19 years from Bihar and Uttar Pradesh respectively have begun childbearing [18]. Moreover, despite the adoption of several policies and programs aimed at improving maternal and child health in the country, poor performance has been recorded in UP and Bihar in antenatal and postpartum care [19, 20].

Teenage pregnancy in India has lifelong and intergenerational health costs, which substantially affect the lives of a major chunk of the adolescent girl population [21] highlighting the necessity for an in-depth examination of the factors determining adolescent pregnancy and pregnancy outcomes. Higher rates of adolescent pregnancy are attributable to a deeply entrenched practice of child marriage, poor access to health care, poverty, and low literacy levels in India [22] and are documented to have major implications on the ill-health of women, educational opportunities and population growth [23]. Despite regionally highly variable rates of adolescent pregnancies there is no sufficient epidemiological evidence in the case of India. Further, insights into the correlates of adolescent pregnancy would help in policy formulation and in providing programmatic response while addressing adolescent pregnancies and progress monitoring towards achieving targets 3.1 and 3.7 of Sustainable Development Goals (SDG) [24]. Keeping in view the poor maternal and child health outcomes and higher adolescent populations in these two Indian states, this study aimed to investigate the contextual factors determining adolescent pregnancy, motherhood, and adverse pregnancy outcomes among adolescents from Uttar Pradesh and Bihar states.

### Data Source

**Fig. 1:**
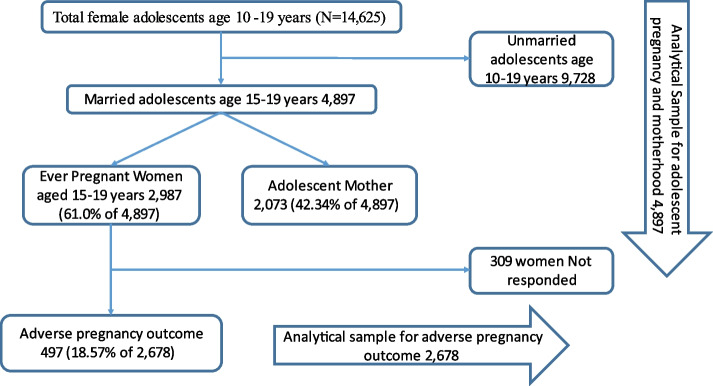
Flow diagram depicting the sample selection process from the UDAYA data

This study uses data collected by the Population Council’s “Understanding the Lives of Adolescents and Young Adults” (UDAYA) project survey, which was conducted in two Indian states, Uttar Pradesh and Bihar, in 2016 under the supervision of the Ministry of Health and Family Welfare. To obtain estimates for the states as a whole, as well as the urban and rural areas of the states, the survey used a multi-stage systematic sampling design [25, 26]. The study’s effective sample size was 4897 married adolescent girls (1732 in UP & 3165 in Bihar) between the ages of 15 and 19. The Population Council’s Institutional Review Board gave its approval to the project and its data collection. It also guaranteed that the participants’ privacy was protected and that informed consent was obtained from respondents during the survey. More information on the sample design, data collection etc. can be found in the UDAYA report [25, 26]. The sample selection criteria have been summarized in Fig. 1. The Population Council’s Institutional Review Board gave its approval to the project and its data collection. It also guaranteed that the participants’ privacy was protected and that informed consent was obtained from respondents during the survey.

### Variable Description

#### Outcome Variables

The variable name pregnancy hood was made by the variable ever been pregnant. If the respondent ever became pregnant it was recoded as 1 means “yes” otherwise 0 means “no”. The second outcome variable was adolescent motherhood. The married adolescent who has given birth to any child before the date of the survey were coded as 1 means “yes” otherwise 2 means “no”. Additionally, the last explanatory variable was formed using the following question 1) Have you ever had a pregnancy, which ended in a stillbirth? (yes/no) 2) Have you ever had a pregnancy that ended in a miscarriage? (yes/no) 3) Have you ever aborted a pregnancy? (yes/no). The response to the above question was coded as 1 means “yes” and 2 means “no”. The variable ‘adverse pregnancy outcome’ was generated using the above three questions. If the respondent has faced any of the problems mentioned above, then it was recoded as 1 “yes” otherwise 0 “no”.

#### Predictor Variables

Married adolescent females (15–19 years) were chosen as the study sample in order to satisfy the objectives. The study was conducted in Bihar and Uttar Pradesh, and respondents’ residences were classified as rural or urban, according to the survey. Caste was recoded as SC/ST (Schedule Caste/ Schedule Tribe), OBC (Others Backward Classless) and Others (General, or Non SC/ST/OBC). Religion was recoded as Hindu and Non-Hindu. The wealth index has been recoded into the following categories: poorest, poorer, middle, richer, and richest [25, 26]. The years of education of eligible respondents were classified as “0-7 years of schooling,” “8-10 years of schooling,” “11-12 years of schooling,” and “12+ years of schooling. “The types of violence experienced by married adolescent women were classified as emotional, physical, and sexual. If the respondent was ever humiliated in front of others or threatened by her spouse to hurt or harm something close to her, the violence was classified as emotional. If the respondent was ever compelled to have sex by her husband, including her first-time sex with him, the violence was classified as sexual. Further, the age gap between the spouses was recoded as < 5 years, 5–10 years, and > 10 years. In addition, the age at marriage and cohabitation were recoded as < 18 and 18–19 years, respectively. Other variables are described in Table 1.

**Table 1:** Characterization of the variables used in this study

Variables	Questions	Response	Recode
**Physical Violence**	Do/did your husband ever do any of the following to you: slap you?	Yes	Yes
	Twist your arm or pull your hair?	No	No
	Push you, shake you, or throw something at you?		
	Punch you with his fist or with something that could hurt you?		
	Kick you, drag you or beat you up?		
	Try to choke you or burn you on purpose?		
	Threaten or attack you with a knife, gun or any other weapon?		
**Substance use (respondent)**	Have you ever consumed tobacco products, eg., smoke cigarette, eat paan, gutka etc.?	Yes	Yes
	Have you ever had alcohol? Have you ever used drugs such as ganja, charas, brown sugar, cocaine or locally made substance eg. Sulochon?	No	No
**Dowry related abuse**	Has anybody in your husband’s family ever said that the dowry/gift/cash you brought was too little	Yes	
	Has anyone in your husband’s family ever asked you to bring more cash/gifts/dowry from your parent’s family?	No	
**Media Exposure**	How often do you watch television? Would you say almost every day, at least once a week, at least once a month, rarely or not at all?	Almost every day	Frequent
	How often do you read a newspaper/ magazine/story books/novels etc.? Would you say almost every day, at least once a week, at least once a month, rarely or not at all?	At least once	
		a week	
	How often do you listen to radio? Would you say almost every day, at least once a week, at least once a month, rarely or not at all?	At least once a month	Rare
	How often do you watch films? Would you say almost every day, at least once a week, at least once a month, rarely or not at all?	Rarely	

#### Analytical Approach

Bivariate analysis was carried out to access the sample characteristics of currently married adolescents and the percentage distribution of pregnancy hood, motherhood and ever-having adverse pregnancy outcome among adolescents in the reproductive age group 15-19 years. A Chi-square test of significance has been used to show the association between pregnancy hood, motherhood, ever adverse pregnancy outcomes with background characteristics. Further for analyzing the significant predictors’ a Multivariable logistic model was used since the dependent variable was nominal with two categories namely no and yes (coded as 0 = No and 1 = Yes), and there was more than one independent variable. Further, equation of the Multivariable logistic regression model is expressed as

$$\log\ \left(\pi /1-\pi \right)=\beta 0+\beta 1X1+\beta 2X2+\dots \beta mXm$$

*Where* ***,*** π indicates the probability of occurrence of an event (e.g., pregnancy, childbirth, stillbirth, miscarriage, abortion), βi indicates the regression coefficient associated with the reference group and Xi indicates the explanatory variables [27, 28]. All of the Statistical analysis included in the study was done using Stata 16 software [29].

## Results

**Fig. 2:**
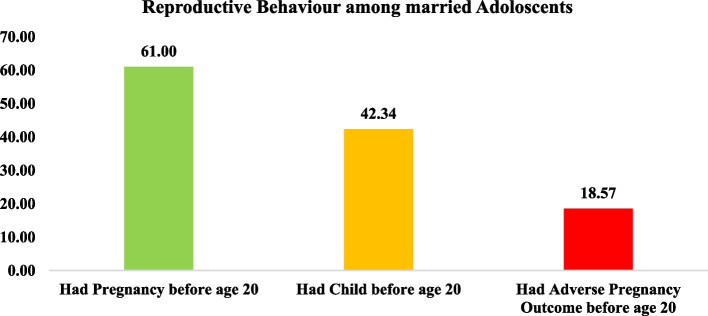
Percentage distribution of pregnancy, motherhood and adverse pregnancy outcome among adolescents in Uttar Pradesh and Bihar (n_1_ = 4897, n_2_ = 2678). Note: n_1_ is the sample size for being pregnant and child before age 20, n_2_ is the sample size for having adverse pregnancy outcome before age 20

Figure 2 displays the percentage distribution of adolescent pregnancy, motherhood, and adverse pregnancy outcome. About three-fifths of the sample population became pregnant before age 20. Among all currently married adolescents, 42% had given birth before age of 20 years. Around 19% of adolescents had an adverse pregnancy outcome (miscarriage, stillbirth, or abortion).

Sample characteristics of currently married adolescents in the reproductive age group 15–19 years are presented in Table 2**.** Overall, 85% of adolescents lived in rural areas. About three-fifths (60%) of the currently married adolescent were from OBC followed by SC/ST (30%) and Others (11%) respectively. The majority of adolescents followed Hinduism (84%) followed by other religions. Approximately half of the sample population had 8 or more years of schooling. Furthermore, very few proportions of women were from the richest wealth quantile (14%) and the majority were poor. About 57% of the adolescent had a spousal age gap of more than 5 years. More than four-fifths of adolescents were married before the legal age of marriage (below 18 years) and nearly three fourth (74%) cohabited with a spouse before the age of 18 years. Around two-thirds of women experienced Sexual violence (65%) while a quarter of respondents experienced emotional violence (29%) and physical violence (26%). Only one-third of the total sample adolescent had frequent media exposure.

**Table 2:** Sample characteristics of currently married adolescent in Uttar Pradesh and Bihar (*n* = 4897)

Sample Variables	Sample	Percentage
**State**		
Uttar Pradesh	1732	35.38
Bihar	3165	64.62
***Place of Residence***		
Urban	722	14.75
Rural	4175	85.25
***Caste***		
SC/ST	1445	29.51
OBC	2920	59.63
Others	532	10.86
***Religion***		
Hindu	4122	84.17
Non-Hindu	775	15.83
***Wealth Index***		
Poorest	848	17.31
Poorer	1047	21.38
Middle	1186	24.22
Richer	1134	23.17
Richest	682	13.93
**Respondent level of education**		
0–7 year of schooling	2486	50.77
8–10 year of schooling	1684	34.38
11–12 year of schooling	595	12.15
12 + year of schooling	132	2.71
**Spousal Age Gap**		
< 5 years	2534	57.14
5-10 years	1753	39.53
> 10 years	148	3.33
**Age at Marriage**		
< 18 years	4109	83.90
18-19 years	788	16.10
**Age at Cohabitation with Spouse**		
< 18 years	3615	73.95
18-19 years	1274	26.05
**Substance use (respondent)**		
No	4744	96.87
Yes	153	3.13
**Intimate Partner Violence (IPV)**^a^		
***Emotional Violence***		
No	3496	71.43
Yes	1399	28.57
***Physical Violence***		
No	3623	73.99
Yes	1274	26.01
***Sexual violence***		
No	1694	34.60
Yes	3203	65.40
**Media Exposure**		
Rare	3246	66.28
Frequent	1651	33.72
**Dowry Requirement**		
No	3630	74.13
Yes	1267	25.87
Total	4897	100.0

Sample total by different characteristics may not be equal to N due to missing cases

^a^IPV is calculated only for married women

**Fig. 3:**
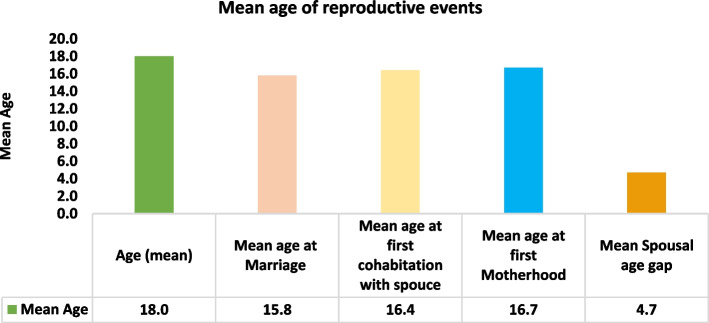
Mean age of specific reproductive events among married Indian adolescents aged 15–19 years in Uttar Pradesh and Bihar (n_1_ = 4897, n2 = 2202, n3 = 4434). Note: n_1_: Sample size for mean age of respondents, mean age at marriage and mean age at first cohabitation with spouse, n_2_: sample size for mean age at first motherhood, n_3_: sample size for mean spousal age gap

Figure 3 shows the mean age of some events such as the mean age of the study population, mean age of marriage, mean age of the first cohabitation with spouse, mean age of the first otherhood and mean age of spousal age gap. The mean age of married adolescents for discussed above events was found as follows 18 years for total married adolescents, 15.8 years for marriage, 16.4 years for first cohabitation, 16.7 years for first motherhood and 4.7 years for spousal age gap.

The association of sociodemographic and behavioral characteristics with adolescent pregnancy, motherhood, and adverse pregnancy outcome is shown in Table 3**.** Pregnancy and motherhood show a significant association with background characteristics such as state, wealth index, respondent level of education, spousal age gap, age at marriage, age at cohabitation with the spouse, dowry-related abuse, and emotional, physical, and sexual violence. Having an adverse pregnancy outcome is found to be significantly associated with the state, spousal age gap, substance use by the respondent and physical violence. The prevalence of adolescent pregnancy and adolescent motherhood is found to be higher in Bihar (64% & 47%). In addition to previous evidence, women from Uttar Pradesh have a higher prevalence (25%) for adverse pregnancy outcomes. The prevalence of dependent variables does not vary much by place of residence, caste and religion. Nearly half of the adolescents became a mother in the poorest wealth quantile and the proportion declined with richer wealth quintiles. With higher educational attainment, the proportion of women experiencing motherhood, getting pregnant and having an adverse pregnancy outcome declined substantially. Women having a husband with more than 10 years of the gap had higher rates for pregnancy hood (75.06%) as well as for motherhood (63.4%) but less prevalent for adverse pregnancy outcomes (11.65%). Additionally, all those adolescents who married and cohabited with a spouse before the age of 18 years and reported being demanded more dowry had higher pregnancy rates and experienced motherhood.

**Table 3:** Association of socio-demographic, personal and behavioural factors with pregnancy, motherhood and adverse pregnancy outcomes among adolescents in Uttar Pradesh and Bihar (n_1_ = 4897, n_2_ = 2678)

Variables	***Pregnancy (%)***	***P***-Value	***Motherhood (%)***	***P***-Value	***Adverse Pregnancy Outcome*** (%)	***P***-Value
***State***						
Uttar Pradesh	56.18	0.000	33.47	0.000	25.38	0.000
Bihar	63.64		47.19		15.43	
***Place of Residence***						
Urban	62.39	0.003	40.64	0.269	18.41	0.437
Rural	60.76		42.63		18.6	
***Caste***						
SC/ST	62.61	0.202	44.85	0.029	18.73	0.175
OBC	60.87		41.97		18.14	
Others	57.35		37.56		20.66	
***Religion***						
Hindu	61.11	0.881	42.83	0.259	17.9	0.261
Non-Hindu	60.42		39.72		22.18	
***Wealth Index***						
Poorest	63.69	0.017	48.25	0.000	18.84	0.866
Poorer	61.87		43.71		20.6	
Middle	59.39		42.95		17.31	
Richer	62.63		40.56		17.69	
Richest	56.42		34.78		18.64	
**Respondent level of education**						
0–7 year of schooling	60.98	0.000	44.46	0.000	19.38	0.155
8–10 year of schooling	63.07		40.14		19.17	
11–12 year of schooling	58.07		42.11		14.15	
12 + year of schooling	48.36		31.38		12.69	
**Spousal Age Gap**						
< 5 years	57.81	0.000	38.45	0.000	19.8	0.022
5-10 years	65.84		46.9		18.57	
> 10 years	75.06		63.4		11.65	
**Age at Marriage**						
< 18 years	65.16	0.000	48.18	0.000	18.28	0.031
18-19 years	39.33		11.87		22.08	
**Age at Cohabitation with Spouse**						
< 18 years	66.78	0.000	51.46	0.000	18.51	0.469
18-19 years	45.12		16.8		18.93	
**Substance Abuse**						
No	60.9	0.448	42.43	0.385	18.12	0.008
Yes	64.16		39.52		31.19	
**Intimate Partner Violence (IPV)**^a^						
***Emotional Violence***						
No	57.4	0.000	37.32	0.000	18.28	0.569
Yes	70.09		54.94		19.11	
***Physical Violence***						
No	56.79	0.000	37.15	0.000	16.39	0.001
Yes	72.99		57.09		22.9	
***Sexual violence***						
No	60.09	0.000	40.05	0.000	17.7	0.339
Yes	62.74		46.66		20.06	
**Media Exposure**						
Rare	60.86	0.25	43.29	0.483	18.85	0.563
Frequent	61.3		40.47		18.02	
**Dowry related abuse**						
No	60.22	0.000	41.06	0.000	17.99	0.417
Yes	63.26		46.01		20.09	

n_1_ is the sample size for being pregnant and child before age 20, n_2_ is the sample size for having adverse pregnancy outcome before age 20, ^a^ IPV is calculated only for married women

Table 4 shows the multivariable logistic regression estimates for adolescent pregnancy, motherhood and adverse pregnancy outcome in India. Results show that with increasing women’s age the odds of pregnancy increased by 2.42 times (OR: 2.42, CI: 2.24–2.62, *p < 0.001*) and motherhood by 2.74 times (OR: 2.74, CI: 2.52–2.9, *p < 0.001*). The risk of experiencing pregnancy and motherhood was 1.32 times and 1.45 times higher in Bihar in comparison to UP(*p < 0.001*). Whereas Bihar shows 46% less (OR: 0.54, CI: 0.43–0.67, *p < 0.001*) likelihood of adverse pregnancy outcome in comparison to UP. The women with 0–7 and 8–10 years of schooling were 69 and 85% more likely to be pregnant in comparison to women with 12 and more years of schooling *(p < 0.001)*. The odds of pregnancy and motherhood increased significantly with the increasing spousal age gap. Adolescents having a spousal age gap of 5 to 10 years were 52% more likely of being pregnant and with more than 10 years of spousal age gap show a 99% (OR: 1.99, CI: 1.35–2.95, *p < 0.001*) higher likelihood of being pregnant. Similarly, adolescents with more than 10 years of spousal age gap were 2.54 times more likely of being a mother. Whereas the spousal age gap was negatively associated with adverse pregnancy outcomes *(p < 0.05)*. For instance, respondents with more than 10 years of spousal age gap were 48% less likely of adverse pregnancy outcomes. Further, the odds of pregnancy and motherhood increased by 1.79 times (OR: 1.79; CI: 1.39–2.30, *p < 0.001*) and 3.21 times (OR: 3.21; CI: 2.33–4.43, *p < 0.001*) among adolescents who married before the age of 18 years than those who married after 18 years. Similarly, adolescents having cohabited with their husbands before age 18 were 3.40 times and 5.75 times more likely of being pregnant and experiencing motherhood. Emotional and physical violence were found to be positively and significantly associated with pregnancy and motherhood. For instance, the women experiencing emotional violence were 1.43 times (OR: 1.43, CI: 1.16–1.76, *p < 0.001*) more likely of being pregnant and 54% more (OR: 1.54, CI: 1.26–1.88, *p < 0.001*) likelihood of being a mother, while in case of physical violence, women were 1.26 times (OR: 1.26, CI: 1.02–1.54, *p < 0.05*) more likely of being pregnant and 1.26 times (OR: 1.26, CI: 1.03–1.54, *p < 0.05*) more likely of being a mother. The risk of adverse pregnancy outcomes was found to be significantly higher for women experiencing physical violence (OR: 1.41, CI: 1.08–1.84, *p < 0.05*). Surprisingly, place of residence, caste, religion, wealth index, substance abuse, sexual violence, media exposure and dowry requirement did not show any significant association with any of these events (pregnancy hood, motherhood, adverse pregnancy outcome).

**Table 4:** Results from Multivariable logistic regression analysis of socio-demographic, personal and behavioural factors with pregnancy, motherhood and adverse pregnancy outcomes among adolescents (n1 = 4426, n2 = 2455)

Variables	Pregnancy	Motherhood	Adverse pregnancy outcome
	Odds Ratio (95% CI)	Odds Ratio (95% CI)	Odds Ratio (95% CI)
***Age***			
15 Years			
16–19 Years	2.42*** [2.24,2.62]	2.74*** [2.52,2.98]	1.01 [0.90,1.14]
***State***			
Uttar Pradesh			
Bihar	1.32*** [1.14,1.54]	1.45*** [1.24,1.70]	0.54*** [0.43,0.67]
***Place of Residence***			
Urban			
Rural	0.91 [0.78,1.07]	1.01 [0.86,1.19]	0.96 [0.76,1.21]
***Caste***			
Others			
SC/ST	1.09 [0.85,1.42]	1.08 [0.82,1.41]	0.93 [0.64,1.37]
OBC	1.01 [0.80,1.27]	0.96 [0.75,1.22]	0.88 [0.62,1.24]
***Religion***			
Hindu			
Non-Hindu	1.06 [0.86,1.29]	1.04 [0.84,1.28]	1.05 [0.78,1.41]
***Wealth Index***			
Richest			
Poorest	1.29 [0.96,1.73]	1.24 [0.91,1.67]	0.91 [0.59,1.39]
Poorer	1.05 [0.80,1.38]	0.97 [0.73,1.29]	0.98 [0.65,1.48]
Middle	1.06 [0.83,1.34]	1.16 [0.90,1.49]	0.82 [0.56,1.19]
Richer	1.13 [0.90,1.41]	1.06 [0.84,1.34]	0.94 [0.67,1.33]
***Respondent level of education***			
12 + year of schooling			
0–7 year of schooling	1.69* [1.13,2.51]	1.38 [0.86,2.20]	1.53 [0.69,3.37]
8–10 year of schooling	1.85** [1.25,2.75]	1.33 [0.84,2.12]	1.54 [0.70,3.39]
11–12 year of schooling	1.23 [0.81,1.86]	1.26 [0.77,2.04]	1.10 [0.48,2.54]
***Spousal Age Gap***			
< 5 years			
5-10 years	1.52*** [1.31,1.76]	1.54*** [1.33,1.78]	0.90 [0.73,1.11]
> 10 years	1.99*** [1.35,2.95]	2.54*** [1.73,3.71]	0.52* [0.29,0.95]
***Age at Marriage***			
18-19 years			
< 18 years	1.79*** [1.39,2.30]	3.21*** [2.33,4.43]	0.55* [0.32,0.94]
***Age at Cohabitation with Spouse***			
18-19 years			
< 18 years	3.40*** [2.72,4.25]	5.75*** [4.51,7.32]	1.45 [0.94,2.24]
***Substance use***			
No			
Yes	0.97 [0.65,1.43]	0.71 [0.48,1.05]	1.14 [0.68,1.89]
**Intimate Partner Violence (IPV)** ^a^			
***Emotional Violence***			
No			
Yes	1.43*** [1.16,1.76]	1.54*** [1.26,1.88]	0.87 [0.65,1.15]
***Physical Violence***			
No			
Yes	1.26* [1.02,1.54]	1.26* [1.03,1.54]	1.41* [1.08,1.84]
***Sexual violence***			
No			
Yes	0.94 [0.80,1.10]	0.98 [0.83,1.16]	1.07 [0.85,1.36]
***Media Exposure***			
Rare			
Frequent	1.12 [0.94,1.32]	1.09 [0.91,1.29]	0.88 [0.68,1.13]
***Dowry related abuse***			
No			
Yes	1.08 [0.91,1.29]	1.07 [0.90,1.27]	1.12 [0.88,1.43]

* *p* < 0.05, ** *p* < 0.01, *** *p* < 0.001, n_1_ is the sample size for being pregnant and child before age 20, n_2_ is the sample size for having adverse pregnancy outcome before age 20, ^a^ IPV is calculated only for married women

## Discussion

Adolescent pregnancy is a major global health burden that can lead to adverse pregnancy and perinatal outcomes. Analyzing the factors associated with pregnancy and adverse pregnancy outcomes among married adolescents is crucial to inform public policies to meet the objectives of SDGs. Adolescent pregnancy in Uttar Pradesh and Bihar is an important issue as these two states have the highest proportion of young age population in the country. Analysis indicates that despite a substantial decline in the overall fertility in the country [18], adolescent pregnancy, motherhood and adverse pregnancy outcomes are still prevalent in the states of UP and Bihar. A major proportion of married adolescents (61%) got pregnant before the age of 20 years and around 42% of all adolescent married women gave birth to a child before reaching the age of 20 years. Additionally, around one-fifth (18.57%) of the eligible sample experienced an adverse pregnancy outcome (miscarriage, stillbirth or abortion) before the age of 20 years. Research suggests an independent association between adolescent pregnancy and increased risks of adverse pregnancy outcomes [11]. Early pregnancy has been documented to possess health risks and these risks are further exacerbated by poverty and inadequate access to maternal and child health care [30]. The mean age of marriage and motherhood for the first time was found to be 15.8 years and 16.7 years respectively which is substantially lower than the minimum legal age at marriage (18 years) and safe motherhood (21 years) respectively. Adolescent motherhood has adverse health effects and socio-economic consequences for the child as well as the mother [14, 31]. Studies report a higher risk of death to be associated with adolescent pregnancy than with adults [32].

Our study findings from multivariable logistics regression analysis show that state, respondent’s level of education, spousal age gap, age at marriage, age at cohabitation with the spouse, and violence (Emotional and physical) were found to be statistically significantly associated with experiencing pregnancy, motherhood and having an adverse pregnancy outcome. A large body of literature has reported an inverse association between women’s educational attainment and adolescent motherhood [33, 34]. The possible explanation for this inverse association is inadequate knowledge of the high-risk fertility period, and less awareness of family planning [35, 36]. Findings from a study by Nahar and Min reveal that women’s higher educational attainment and higher age at marriage results in postponing the age of experiencing pregnancy in Bangladeshi Adolescent women [37]. Another study done in Malaysia reveals that adolescent pregnancy is associated with low education, a child raised by a single parent, low socioeconomic status and substance abuse as well [38]. Surprisingly, the study result also indicates that women who got married before 18 years were 45% less likely to have an adverse pregnancy outcome in comparison to those who were married at age 18–19 years. Additionally, age at first cohabitation with a spouse is negatively associated with pregnancy, motherhood and as well as adverse pregnancy outcomes. Literature has reported controversial findings regarding the association between marriage before 18 years and adverse maternal and child health outcomes such as pregnancy termination, unintended pregnancy, underutilization of maternal health services, female sterilization, stillbirth/miscarriage, infant mortality, child mortality, malnutrition [39–43]. Hospital-based studies have reported that teenage pregnancy itself is ‘causally’ associated with a poor obstetric outcomes such as perinatal death [44]. Further, no significant adverse pregnancy outcome is observed among adolescent mothers where high-quality maternity care is available [45, 46]. Numerous pieces of literature have highlighted the importance of quality maternity care in reducing adverse pregnancy outcomes [45–47]. We did not any significant association between wealth status and pregnancy hood, motherhood and adverse pregnancy outcome. However, it’s documented that lower educational status and wealth index results in low nutrient intake that can cause adverse pregnancy outcomes [48].

Considering the higher number of adolescent pregnancies and early motherhood, it is likely that the pregnancy might be unintended. Since, among disadvantaged groups, knowledge awareness and access to contraceptives often lead to unplanned pregnancies. Thus, it is important to aim at improving adolescent education on sexual and reproductive rights and access to preferred contraceptive methods [33]. Consistent with our findings, Islam and colleagues found that a higher spousal age gap was associated with higher adolescent motherhood among adolescents as well as adult women. Unequal power relations and low inter-spousal communication between spouses with a higher age gap renders in lower participation in the decision-making process of the use of contraceptives [49, 50].

In the present study, women who experienced physical violence were at higher risk for having an adverse pregnancy outcome than those who did not experience physical violence. Our results are coherent with several recent studies reporting a similar level of IPV experienced by women having a miscarriage [51, 52]. According to studies, women with undesired or unexpected pregnancies who are in abusive relationships are likely to have a higher rate of induced abortions and a risk factor for unwanted pregnancy [52, 53]. Abortion may be a direct outcome of physical assault-related bodily trauma or an indirect result of relationship stress [54]. Women who experience IPV have poorer decision-making skills and more unwanted pregnancies, usually because they use less contraception [55]. The unequal position of women in a relationship and patriarchal society and the material dependency of women, lack of alternative opportunities and the socially acceptable norm of female subordination to feudalistic values makes women vulnerable to IPV [56]. Published findings suggest that violence during pregnancy causes physical trauma and stress which adversely affects pregnancy outcomes [57].

## Conclusion

To summarize, our study highlights the high prevalence of adolescent pregnancy and adverse pregnancy outcomes. Adolescent pregnancy and its adverse outcome are further determined by various socio-economic, demographic characteristics and other factors. Thus it is pertinent that addressing poverty, mass media exposure can increase the age at marriage and thus reduce the adverse pregnancy outcomes in the country. Further, violence was found to be a significant factor affecting motherhood and pregnancy outcomes. Additionally, regional and national level efforts focused on improving early marriage, education and empowering women and girls can be beneficial. Moreover, provisions for ensuring women’s rights and the needs of marginalized and vulnerable women should be addressed.

### Limitation

The cause or consequence of an adolescent pregnancy could not be established due to the cross-sectional nature of the data. For examples, it was not possible to estimate the time spent in the union before getting pregnant. Additionally, we did not include access to health services in our analysis which might impact the pregnancy outcome; this highlights the need for further study including access to health services. Since time is an important determinant in examining adolescent motherhood, further research would be more beneficial by taking the “time” effect into account.

## Data Availability

The information was gathered as part of the Population Council’s UDAYA project, which is publicly accessible on the Harvard Dataverse website on request at https://dataverse.harvard.edu/dataset.xhtml?persistentId=doi:10.7910/DVN/RRXQNT and https://dataverse.harvard.edu/dataset.xhtml?persistentId=doi:10.7910/DVN/ZJPKW5.
